# Supplementary material for: Electrically driven lasing from a dual-cavity perovskite device
Source: Nature. 2025 Aug 27;645(8080):369–74. doi: 10.1038/s41586-025-09457-2 (PMC12422965; doi:10.1038/s41586-025-09457-2)
Supplement: Supplementary file 1 — Supplementary Notes 1–3, Supplementary Figs. 1–9 and Supplementary References [file 41586_2025_9457_MOESM1_ESM.pdf]

nature portfolio

https://doi.org/10.1038/s41586-025-09457-2

Supplementary information

Electrically driven lasing from a dual-cavity perovskite device

In the format provided by the authors and unedited

Supplementary Information for

Electrically driven lasing from a dual-cavity perovskite device

Chen Zou[1,2*], Zhixiang Ren[1], Kangshuo Hui[1], Zixiang Wang[1], Yangning Fan[1], Yichen Yang[1], Bo Yuan[1], Baodan Zhao[1,2*], Dawei Di[1,2,3*]

1. State Key Laboratory of Extreme Photonics and Instrumentation, College of Optical Science and Engineering, International Research Center for Advanced Photonics, Zhejiang University, Hangzhou, China.
2. ZJU-Hangzhou Global Scientific and Technological Innovation Center, Zhejiang University, Hangzhou, China.
3. ZJU-UIUC Institute, Zhejiang University, Haining, China.

*Corresponding authors, Email: daweidi@zju.edu.cn (D.D.); zouchen@zju.edu.cn (C.Z.); baodanzhao@zju.edu.cn (B.Z.).

Table of Contents

Supplementary Note 1 | The optical cavity length of microcavity I.

Supplementary Note 2 | Estimation of excitation densities in microcavity II under pulsed and CW optical pumping.

Supplementary Note 3 | Analysis of response time for microcavity PeLEDs.

Supplementary Fig. 1 | Reflectance spectrum of the semi-transparent Au electrode in microcavity I.

Supplementary Fig. 2 | Optical microscope image of an $FAPbI_3$ perovskite single crystal.

Supplementary Fig. 3 | Reflectance spectrum of optimized DBR3.

Supplementary Fig. 4 | EL intensity profile of a microcavity PeLED.

Supplementary Fig. 5 | Polarisation plots for different lasing spots in microcavity II under optical pumping.

Supplementary Fig. 6 | Polarisation plots of electrically-driven dual-cavity perovskite lasers, showing device-to-device variations in the orientations of polarisation.

Supplementary Fig. 7 | Effects of emission directionality and inter-cavity distance on coupling efficiency between microcavity I and microcavity II.

Supplementary Fig. 8 | Transient PL decay curves at different current densities for a microcavity PeLED with an active area of 0.02 mm^2.

Supplementary Fig. 9 | Differential resistance and transient EL of the microcavity PeLED sub-unit.

Supplementary References

Supplementary Note 1 | The optical cavity length of microcavity I

The functional layers within microcavity I (between DBR and Au) are ITO (~110 nm), ZnO (~20 nm), $Cs_{0.5}FA_{0.5}PbI_2Br$ perovskite (~25 nm), TFB (~30 nm), and MoO_3 (~7 nm). The refractive indices of these layers are ~1.68, ~2.0, ~2.4, ~1.7 and ~2.3, respectively[1]. The optical cavity length inside the microcavity (L_{in}) could be obtained using

$$L_{in} = \Sigma n_i d_i \quad \text{(S1-1)}$$

where n_i, d_i are the refractive index and thickness of each layer inside the microcavity. Using this equation, L_{in} is calculated to be 352 nm, which is close to $\lambda_0/2$ ($\lambda_0 = 680\ nm$). Such a cavity length is sufficiently long for the emission at ~680 nm.

Further, we consider the possibility of forming an extended cavity, taking into account the penetration of optical fields into the DBRs. The penetration distance into the DBRs (D_{DBR}) can be estimate using[2]

$$D_{DBR} = \frac{\lambda_0}{2}\frac{n_1 n_2}{n_c(n_2 - n_1)} \quad \text{(S1-2)}$$

where n_1 and n_2 are the refractive indices of the two alternating materials in the DBR. n_c (1.83) is the effective refractive index inside the cavity. Using this equation, D_{DBR} is calculated to be 697 nm. n_{DBR} is the effective refractive index of the DBR and it can be obtained through

$$n_{DBR} = \frac{n_1 d_1 + n_2 d_2}{d_1 + d_2} \quad \text{(S1-3)}$$

where d_1 and d_2 are the thicknesses of the two alternating materials in the DBR. Using this equation, n_{DBR} is calculated to be 1.88.

The total extended cavity length (L_{ext}) equals the cavity length inside the cavity (L_{in}) plus the length of optical penetration into the DBRs.

$$L_{ext} = L_{in} + n_{DBR} D_{DBR} \quad \text{(S1-4)}$$

Using this equation, L_{ext} is estimated to be 1662 nm, which is close to $\frac{5}{2}\lambda_0$. Therefore, constructive optical interference at λ_0=680 nm can be established with such a cavity configuration, regardless of whether the effective cavity length is considered to be within the cavity (L_{in}) or extended into the DBR (L_{ext}). The cavity mode at around 680 nm can be supported by the microcavity.

Supplementary Note 2 | Estimation of excitation densities in microcavity II under pulsed and CW optical pumping

From our pulsed optical pumping experiments, we estimate the excitation density (n) in the single-crystal perovskite microcavity sub-unit (microcavity II) based on the following equation[3]:

$$n = \frac{\alpha P}{h\nu d} \qquad \text{(S2-1)}$$

where P is the excitation fluence, $h\nu$ is the excitation photon energy (3.1 eV), d is the thickness of the perovskite single crystal (~180 nm), and α is the optical absorption (~75%) obtained by considering the measured reflection and transmission at the excitation wavelength. The lasing threshold of microcavity II is found to be 0.47 μJ cm^{-2} under femtosecond laser pumping (Extended Data Fig. 8a). According to the equation above, the excitation density at the threshold is estimated to be 3.9×10^{16} cm^{-3}. The PL decay kinetics of the perovskite single crystals were measured under different excitation densities (Extended Data Fig. 8b). The carrier lifetimes were found to be ~134.7 ns at the excitation density (3.9×10^{16} cm^{-3}, as estimated above) corresponding to the lasing threshold (0.47 μJ cm^{-2}).

Next, we estimate the CW threshold of optically pumped lasing from microcavity II through the following equation[4]:

$$P_{th,CW} \cdot \tau = F_{th} \qquad \text{(S2-2)}$$

where F_{th} is the pulsed threshold (0.47 μJ cm^{-2}), $P_{th,CW}$ is the CW threshold (W cm^{-2}), and τ is the carrier lifetime, which is ~134.7 ns at the excitation density equivalent to the lasing threshold (0.47 μJ cm^{-2}). Using this equation, the CW threshold ($P_{th,CW}$) is estimated to be 3.4 W cm^{-2}, in reasonable agreement with the experimentally measured CW threshold (6.6 W cm^{-2}). In summary, the low CW lasing thresholds are consistent with the low pulsed thresholds and the long carrier lifetimes near the threshold. The low thresholds in turn ensure small excitation densities, leading to longer lifetimes at just below the thresholds.

Supplementary Note 3 | Analysis of response time for microcavity PeLEDs

At above the lasing threshold, the carrier lifetimes in the single-crystal perovskite microcavity sub-unit (microcavity II) are on the order of picoseconds, as this is a typical carrier lifetime for perovskite lasers[5-7]. The overall rise and fall times of the device are on the order of tens of nanoseconds, and are therefore limited by the transient characteristics of the microcavity PeLED sub-unit (microcavity I), whose response time is the sum of the effective carrier lifetime ($\tau_{carrier}$) and the resistance-capacitance (RC) time constant (τ_{RC}), where τ_{RC} is the product of the differential resistance and the parasitic capacitance of the device ($\tau_{RC} = R \cdot C$). The total response time of the device can be written as

$$\tau_{response} = \tau_{carrier} + \tau_{RC} \qquad \text{(S3-1)}$$

Under intense current injection (>200 A cm^{-2}, above the lasing threshold), the measured rise time is ~24.4 ns for the microcavity PeLED with an active area of 0.02 mm^2, which is likely dominated by the relatively large RC constant (τ_{RC}) of the device, considering the spontaneous lifetime of the microcavity PeLED is less than 2 ns under intense current injection (Supplementary Fig. 8). The differential resistance (R) can be obtained by $R=dV/dI$ (Supplementary Fig. 9a). For the microcavity PeLED sub-unit with an active area of 0.02 mm^2, R is estimated to be 243 Ω at a current density of 212 A cm^{-2}. Further, we calculate the capacitance (C) using

$$C = \frac{\varepsilon_r S}{4\pi k d} \qquad \text{(S3-2)}$$

where ε_r is the relative permittivity of perovskite (ε_r=32)[8], S is the facing area of the two plates (0.02 mm^2), d is the distance between the two plates (90 nm), and k is the Coulomb's constant (9.0×10^9 N m^2/C^2). Using this equation, the capacitance is calculated to be 62.9 pF. The RC constant can be found using $\tau_{RC} = R \cdot C$=15.2 ns. The transient EL intensity (L) from the microcavity PeLED sub-unit during the rise stage can be described by[9]

$$L = tanh^2\left(\frac{t}{\tau_{response}}\right) \qquad \text{(S3-3)}$$

where $\tau_{response} \approx \tau_{RC}$, and t is the time after the onset of electrical trigger. Using this equation, we have simulated the transient EL response of the microcavity PeLED (Supplementary Fig. 9b). For a τ_{RC} of 15.2 ns, the simulated rise time of the device is 23 ns, in good agreement with the experimentally measured rise time (24.4 ns) for the dual-cavity device with an active area of 0.02 mm^2 (Supplementary Fig. 9b). The fall time of the EL intensity shows a similar dependence on the RC constants.

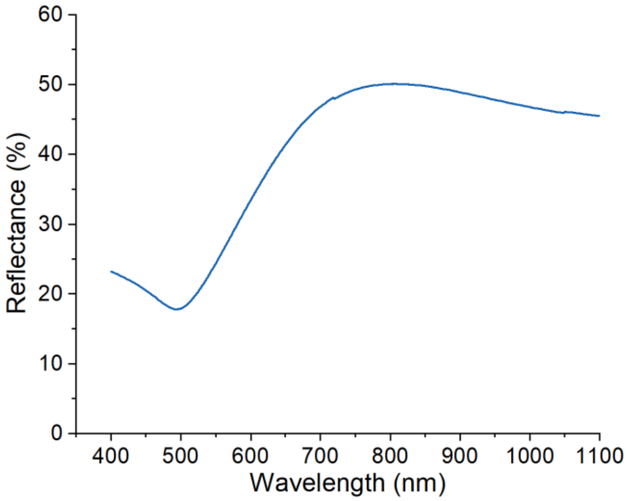

Supplementary Fig. 1 | Reflectance spectrum of the semi-transparent Au electrode in microcavity I.

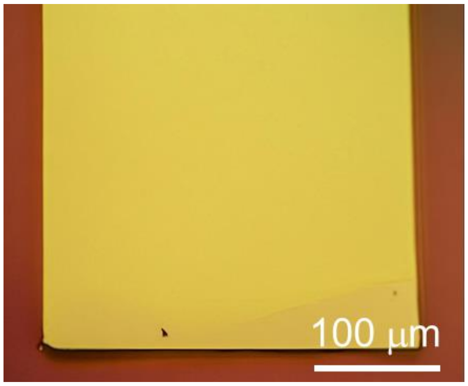

Supplementary Fig. 2 | Optical microscope image of an $FAPbI_3$ perovskite single crystal. Scale bar, 100 µm.

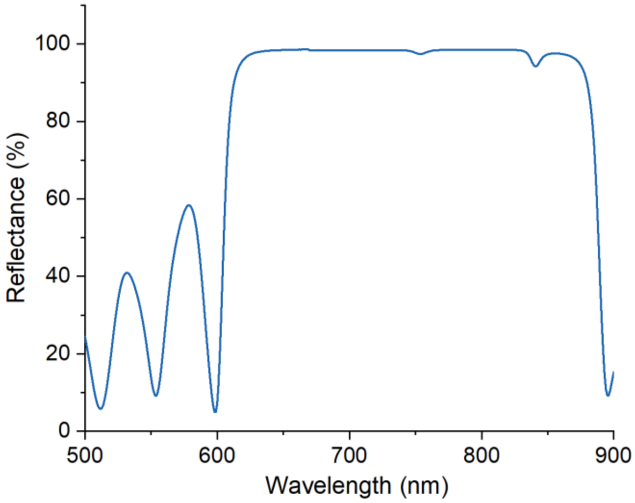

Supplementary Fig. 3 | Reflectance spectrum of optimized DBR3.

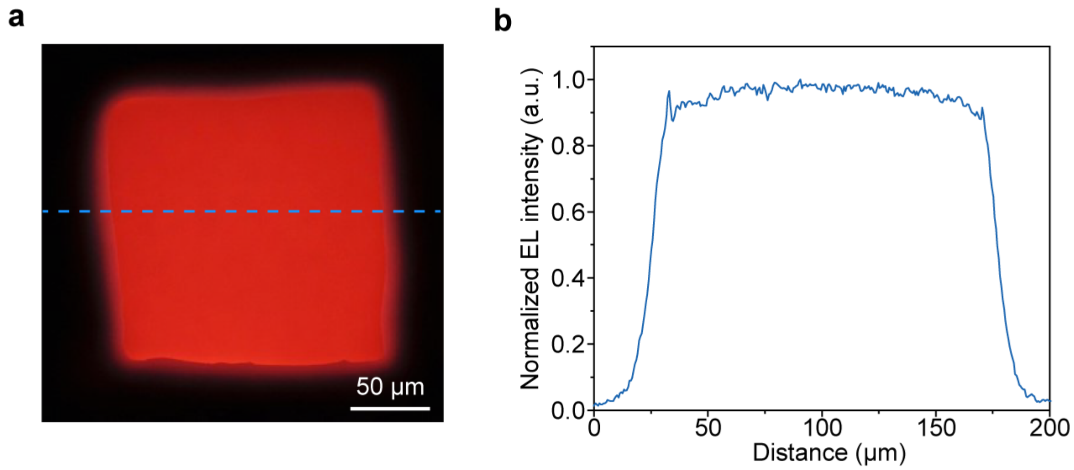

Supplementary Fig. 4 | EL intensity profile of a microcavity PeLED. a, Optical microscopy image (dark-field) of a working microcavity PeLED (active area, 0.02 mm^2). **b**, EL intensity profile extracted along the blue dashed line in **a.**

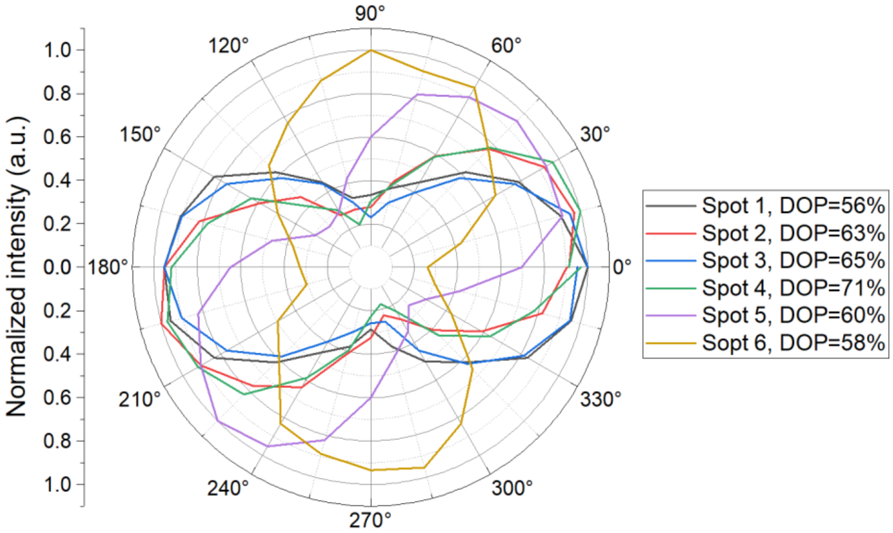

Supplementary Fig. 5 | Polarisation plots for different lasing spots in microcavity II under optical pumping. The lasing spectra were measured with a rotational polariser. The peak lasing intensity as a function of the polariser angle is plotted. DOP refers to degree of polarisation. Microcavity II was pumped by 1-μs optical pulses from an electrically modulated 405-nm continuous-wave (CW) laser at a repetition rate of 10 Hz. The excitation intensity was 13 W cm^{-2} (above the threshold). The diameter of the focused pump spot was ~18 μm.

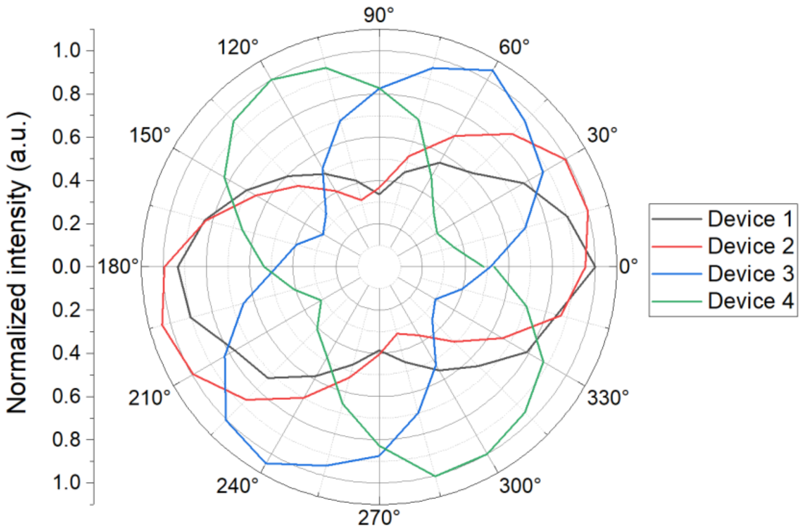

Supplementary Fig. 6 | Polarisation plots of electrically-driven dual-cavity perovskite lasers, showing device-to-device variations in the orientations of polarisation. The devices were driven at a current density of ~280 A cm^{-2} (above threshold).

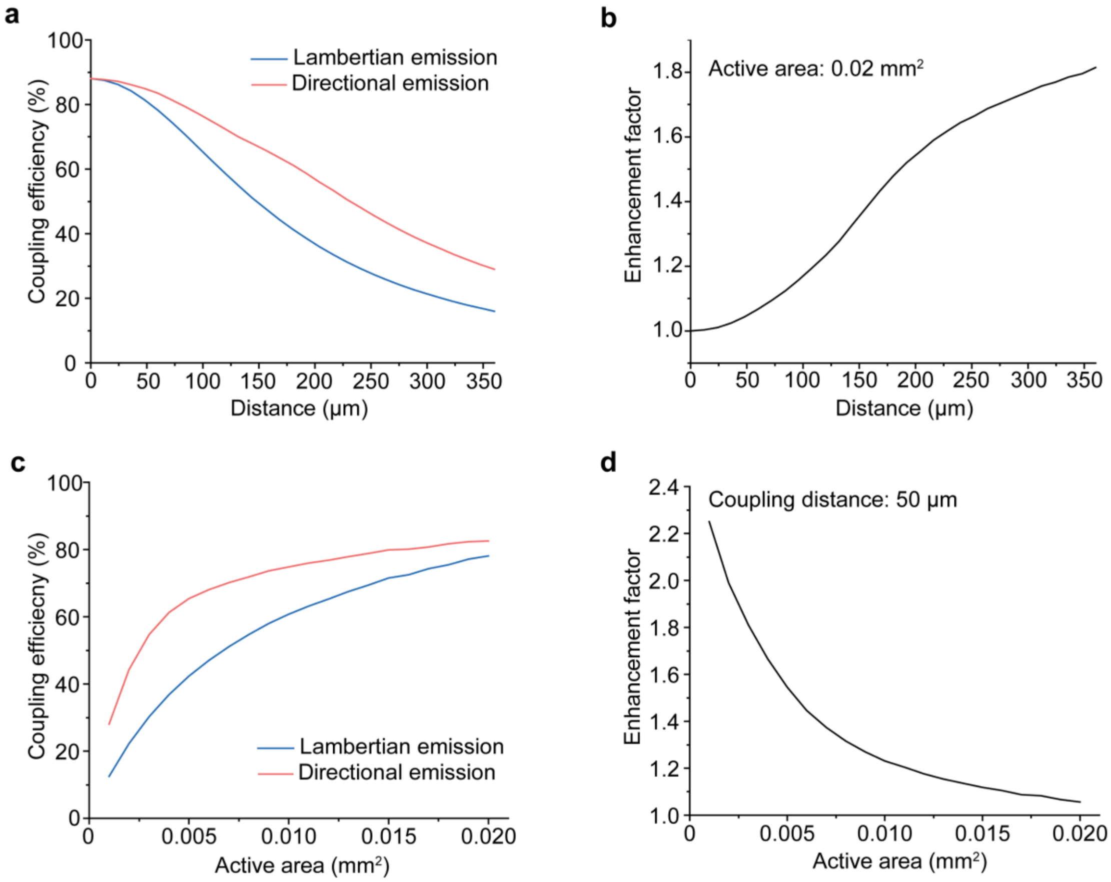

Supplementary Fig. 7 | Effects of emission directionality and inter-cavity distance on coupling efficiency between microcavity I and microcavity II. a, Simulated coupling efficiency versus coupling distance for Lambertian and directional emission. **b**, The enhancement of the coupling efficiency due to emission directionality for different distances. Device area: 0.02 mm^2. **c**, Simulated coupling efficiency versus active area for Lambertian and directional emission, at a fixed coupling distance of 50 μm. **d**, The enhancement of the coupling efficiency due to emission directionality for different active areas, at a fixed coupling distance of 50 μm.

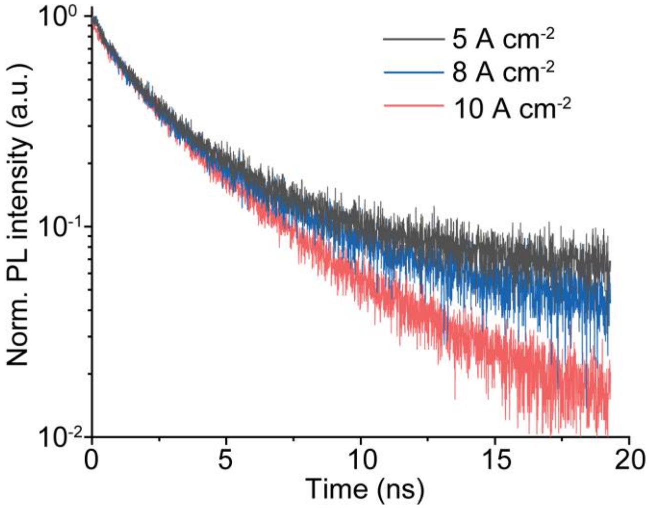

Supplementary Fig. 8 | Transient PL decay curves at different current densities for a microcavity PeLED with an active area of 0.02 mm^2. The microcavity PeLED was excited by a femtosecond laser (excitation wavelength: 400 nm; pulse duration: ~270 fs; excitation fluence: ~1 μJ cm^{-2}), while driven at constant current densities of 5 A cm^{-2}, 8 A cm^{-2} and 10 A cm^{-2}. The spontaneous emission lifetime of the microcavity PeLED is about 2 ns.

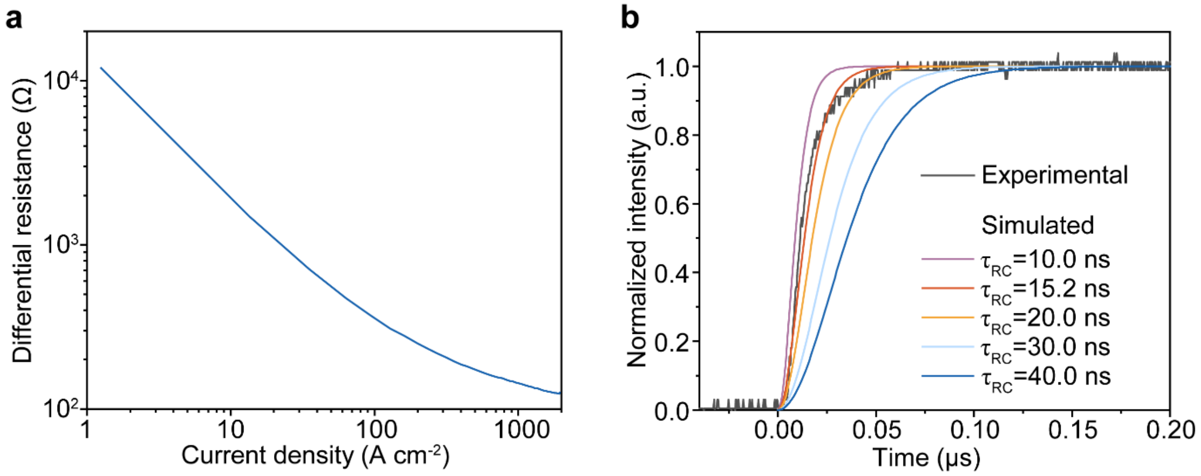

Supplementary Fig. 9 | Differential resistance and transient EL of the microcavity PeLED sub-unit. a, Differential resistance as a function of current density. **b**, Simulated transient EL of microcavity PeLEDs with different RC time constants (using Equation S3-3 in Supplementary Note 3). An RC constant of 15.2 ns (red curve) yields a simulated EL response with a rise time of 23 ns, which closely agrees with the experimental data (rise time: 24.4 ns). Active area of the device: 0.02 mm^2. Current density: ~212 A cm^{-2}.

Supplementary References

1 Shi, X. B. *et al.* Optical energy losses in organic-inorganic hybrid perovskite light-emitting diodes. *Adv. Opt. Mater.* **6**, 1800667 (2018).

2 Bouteyre, P. *et al.* Room-temperature cavity polaritons with 3D hybrid perovskite: toward large-surface polaritonic devices. *ACS Photonics* **6**, 1804-1811 (2019).

3 Enomoto, S. *et al.* Drastic transitions of excited state and coupling regime in all-inorganic perovskite microcavities characterized by exciton/plasmon hybrid natures. *Light Sci. Appl.* **11**, 8 (2022).

4 Brenner, P. *et al.* Continuous wave amplified spontaneous emission in phase-stable lead halide perovskites. *Nat. Commun.* **10**, 988 (2019).

5 Zhao, C. *et al.* High performance single-mode vertical cavity surface emitting lasers based on $CsPbBr_3$ nanocrystals with simplified processing. *Chem. Eng. J.* **420**, 127660 (2021).

6 He, Y. *et al.* Lasing properties and carrier dynamics of $CsPbBr_3$ perovskite nanocrystal vertical-cavity surface-emitting laser. *Nanophotonics* **12**, 2133-2143 (2023).

7 Aoyagi, S. *et al.* Single-crystal $CsPbBr_3$-based vertical cavity surface emitting laser. *Opt. Lett.* **50**, 702-705 (2025).

8 Chiba, T. *et al.* Anion-exchange red perovskite quantum dots with ammonium iodine salts for highly efficient light-emitting devices. *Nat. Photon.* **12**, 681-687 (2018).

9 Bao, H. *et al.* Quantitative determination of charge accumulation and recombination in operational quantum dots light emitting diodes via time-resolved electroluminescence spectroscopy. *J. Phys. Chem. Lett.* **14**, 1777-1783 (2023).
